# Determinants of stunting and severe stunting among under-fives: evidence from the 2011 Nepal Demographic and Health Survey

**DOI:** 10.1186/1471-2431-14-239

**Published:** 2014-09-27

**Authors:** Rina Tiwari, Lynne M Ausman, Kingsley Emwinyore Agho

**Affiliations:** Nutrition Promotion and Consultancy Service, Kathmandu, Nepal; Friedman School of Nutrition Science and Policy, Tufts University, Medford, Massachusetts USA; School of Science and Health, University of Western Sydney, Sydney, New South Wales Australia

## Abstract

**Background:**

Stunting remains a major public health concern in Nepal as it increases the risk of illness, irreversible body damage and mortality in children. Public health planners can reshape and redesign new interventions to reduce stunting and severe stunting among children aged less than 5 years in this country by examining their determinants. Hence, this study identifies factors associated with stunting and severe stunting among children aged less than five years in Nepal.

**Methods:**

The sample is made up of 2380 children aged 0 to 59 months with complete anthropometric measurements from the 2011 Nepal Demographic and Health Survey (NDHS). Simple and multiple logistic regression analyses were used to examine stunting and severe stunting against a set of variables.

**Results:**

The prevalences of stunting and severe stunting were 26.3% [95% confidence Interval (CI): 22.8, 30.1] and 10.2% (95%CI: 7.9, 13.1) for children aged 0–23 months, respectively, and 40.6 (95%CI: 37.3, 43.2) and 15.9% (95%CI: 13.9, 18.3) for those aged 0–59 months, respectively. After adjusting for potential confounding factors, multivariable analyses showed that the most consistent significant risk factors for stunted and severely stunted children aged 0–23 and 0–59 months were household wealth index (poorest household), perceived size of baby (small babies) and breastfeeding for more than 12 months (adjusted odds ratio (AOR) for stunted children aged 0–23 months = 2.60 [95% CI: (1.87, 4.02)]; AOR for severely stunted children aged 0–23 months = 2.87 [95% CI: (1.54, 5.34)]; AOR for stunted children aged 0–59 months = 3.54 [95% CI: (2.41, 5.19)] and AOR for severely stunted children aged 0–59 months = 4.15 [95% CI: (2.45, 6.93)].

**Conclusions:**

This study suggests that poorest households and prolonged breastfeeding (more than 12 months) led to increased risk of stunting and severe stunting among Nepalese children. However, community-based education intervention are needed to reduce preventable deaths triggered by malnutrition in Nepal and should target children born to mothers of low socioeconomic status.

## Background

Stunting and other effects of under-nutrition increase the risk of illness, irreversible body damage, and increased suboptimal brain development and affect cognitive ability and mortality in children [1]. It is estimated that about 165 million children in many low- and middle-income countries are stunted [2]. Stunting is one of the leading causes of the global burden of disease in childhood and 80% of this burden is in developing countries [3, 4]. Childhood under-nutrition plays an important public health role in monitoring nutritional and health status of the population and survival [5, 6].

Under-nutrition hinders socioeconomic development of a nation [1]. Consequently, its eradication has gained global recognition and sustainable development. In Nepal, stunting remains a serious problem as nearly half (41 per cent) of children aged under five years are stunted [7]. The causes of childhood under-nutrition in Nepal are complex, multidimensional, and interrelated, ranging from fundamental factors such as slow economic growth, to specific factors such as respiratory infection and diarrhoeal diseases [8, 9].

Previous studies in Nepal have found factors such as sex of child, child’s age and birth weight, birth order, number of siblings, wealth index, mother’s education, mother’s body mass index and access to health care to be common determinants of stunting [9, 10]. Additionally, various efforts have been made to reduce under-nutrition in Nepal [11]. Despite these commitments and investment, the prevalence of stunting is still high [7]. In order to reduce the burden of disease on Nepalese children, it is important to target those children who are most at risk, such as stunted children [12–14]. This study utilized the most recent Nepal Demographic and Health Survey (NDHS 2011) data to examine the determinants of stunting and severe stunting among children aged less than five years. Findings from this study can be generalised to cover populations with similar characteristics and would be useful to public health researchers and policy makers in reviewing and designing new intervention strategies aimed at reducing the number of malnourished children. The results will also provide vital information on preventable illnesses and identify where health gains can be made to prevent stunting. The findings may also allow policy-makers to direct resources to the most vulnerable segments of the population, and thus make better use of resources.

### Ethics

The NDHS surveys were approved by Nepal Health Research Council, Nepal and ICF Macro Institutional Review Board in Calverton, Maryland, USA. The NDHS obtained written consent from the respondents. Mothers provided consent of their children to provide the information. For analysis, Principal Investigator (PI) received permission from Macro International online for the use of available dataset. PI also obtained approval from Social, Behavioural and Educational Research, Institutional Review Board, Tufts University as exempt category 4 as defined in 45 CFR 46.101 (b).

## Methods

### Data sources

The data examined were from the 2011 NDHS. This survey was conducted by the Department of Health Services, Ministry of Health and Population in collaboration with USAID. The survey data were a two-stage, stratified, nationally representative sample of households. At the first stage of sampling, 289 Primary Sampling Units (PSUs) (95 sub-wards in urban areas and 194 groups of wards in rural areas) were selected using systematic sampling with probability proportion to size.

In the selection of households, 12,918 women were identified as eligible for the individual interview. A total of 12,674 women aged between 15 and 49 years were interviewed. Out of this number, 3,701 were resident in urban areas and 8,973 were rural dwellers. The interviews yielded a response rate of 98%. The 2011 NDHS collected anthropometric data for all children. Non-biological children of women were included in the interviews. Each team of interviewers carried a scale and measuring board. Measurements were made using lightweight SECA scales (with digital screens) on children with valid dates of birth (month and year) and valid measurement of both height and weight. Recumbent heights were measured for children aged 2 years or younger, or those who were shorter than 85 cm. Standing heights were measured for all other children [7]. This present analysis was restricted to children with complete anthropometric measurements, children aged 0–59 months, and the total weighted sample was 2380.

### Stunting (Height-for-age)

Height-for-age z-scores were used to assess the chronic nutritional status of children under-5 years. This was accomplished by adapting the Child Growth Standards of the World Health Organization (WHO) [15]. The height-for-age *z*-score, as defined by the WHO, expresses a child’s height in terms of the number of standard deviations above or below the median height of healthy children in the same age group or in a reference group. We classified children with a measurement of < −2 SD from the median of the reference group as short for their age (stunted), while children with measurement of < −3SD from the median of the reference group were considered to be severely stunted [16].

### Potential risk factors

The explanatory variables were classified into four levels: parental-, child-, household- and community-level factors. Parental-level factors included maternal working status, maternal education, mother’s age, mother’s age at child birth, mother’s breastfeeding status, duration of breastfeeding, marital status, mother’s literacy, partner’s education, partner’s occupation, birth order, preceding birth interval, type of delivery assistance, antenatal clinic visits, timing of postnatal check-up and place of delivery. Mode of delivery was divided into three categories: delivered at home, delivered at health facility with non-caesarean section, and delivered at a health facility with caesarean section. Child-level factors were: sex of the baby and acute respiratory infection (defined as having symptoms of cough accompanied by short, rapid breathing which was chest related during 2 weeks preceding the survey). Any child with watery or blood and mucus stool in the preceding 2 weeks was considered as having diarrhoea. Household-level factors were household food insecurity, household wealth index, and sources of drinking water; community-level factors were: type of residence, caste group, ecological zone, geographical zones and sub-region. The household food insecurity factor was calculated by summing all the seven household food insecurity (access) frequency questions withscores ranging from 0 to 27. Food secure was a score of 0, and mild (1–2), moderate (3 to 10), and severe (more than 10) food insecurity represented the three groupings [7]. The caste and ethnic group variables were merged into six categories. This was done by merging all Brahmin Chhetris irrespective of their ecological locations into a single category referred to as “Brahmin/Chhetri”. Similarly, the Janajati from Terai was merged with other Terai castes to make a single category and referred to as “TeraiJanajati and other Terai castes” and Dalits from Hill and Terai were also combined as one. Likewise, Muslims and other castes were put together as “Muslims and others” [7].

Household wealth index was calculated as a score of household assets such as ownership of means of transport, ownership of durable goods, and household facilities. These were weighted using the principal components analysis method [17]. This index was divided into five categories (quintiles), and each household was assigned to one of these categories. The bottom 40% of the households was referred to as the poorest and poorer households, the next 20% as the middle-class households, and the top 40% as rich and richest households.

### Statistical analyses

To determine factors associated with stunting and severe stunting in children aged 0–23 months and children 0–59 months – the dependent variable was expressed as a dichotomous variable, i.e. category 0 [not stunted (> − 2SD) or not severely stunted (> − 3SD)] and category 1 [stunted (> − 2SD) or severely stunted (> − 3SD)].

Analyses were performed using Stata version 12.0 (StataCorp, College Station, TX, USA). ‘Svy’ commands were used to allow for adjustments for the cluster sampling design, weights and the calculation of standard errors. The Taylor series linearization method was used in the surveys when estimating confidence intervals (CIs) around prevalence estimates of stunting and severe stunting among children aged 0–59 months. Survey logistic regression was used to adjust for the complex sampling design and weights. First, univariate binary logistic regression analysis was performed to examine the association between stunted and severely stunted children aged 0–23 months and overall stunted children 0–59 months. Second, the factors associated with stunting and severe stunting were examined in a multiple logistic regression model. A stepwise backward elimination approach was applied and collinearity was tested in the final model and reported. The odds ratios with 95% CIs were calculated in order to assess the adjusted risk of independent variables, and those with *P* < 0.05 were retained in the final model.

## Results

### Characteristics of the sample

Of the total sample of 2380 children aged 0–59 months, majority (91.1%) lived in rural areas. More than half (56.6%) of the interviewed mothers were employed in the last 12 months, and 32.6% had secondary or higher level of education. Of the total births, 65.5% took place at home and by non-caesarean section, and 30% (see Table 1) were delivered at the health care facility and by non-caesarean section or vaginal birth. The remaining 4.5% were delivered by caesarean section at the health care facility. In the sample, male and female children were almost equally represented. About 83% of mothers had made at least one antenatal clinic visit during pregnancy and a majority of the mothers in the sample were within 20–29 years of age. Also, approximately 44% of households reported food security and 16% reported severe food insecurity. According to the mothers’ perception, 64.5% of children were average size, 17.7% were small or very small size, and 17.8% were large size at birth. As shown in Table 1, the proportion of mothers who could not read a sentence was 42.7%. Nearly 24% of children lived in the Eastern geographical zones and 31.6%, 18.2%, 15.1% and 11.3% of children lived in Central, Western, Mid-western, and Far-western geographical zones respectively.

**Figure 1:**
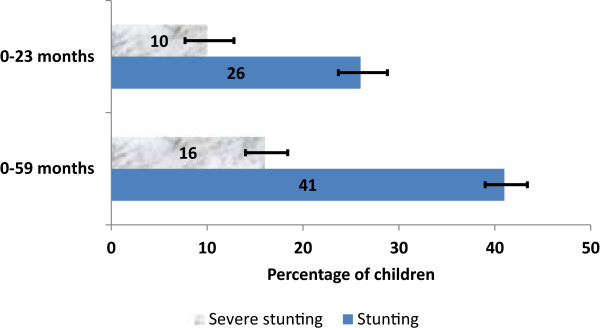
**Prevalence of stunting and severe stunting among children aged 0-59 months.**

As shown in Figure 1, the prevalence of stunted children aged 0–23 months was 26% and a higher 41% for children aged 0–59 months. The overall prevalence of severely stunted children aged 0–23 months and 0–59 months were 10% and 16%, respectively.

**Table 1:** **Characteristics of parental-, child-, household- and community-level factors of stunted children aged 0–59 months in Nepal 2011**

Characteristic	n	%
***Parental factors***		
**Maternal working status**		
Non-working	1033	43.4
Working (past 12 months)	1347	56.6
**Maternal education**		
No education	1128	47.4
Primary	469	19.7
Secondary and above	782	32.9
**Partner’s occupation**		
Non agriculture	1679	70.6
Agriculture	593	24.9
Not working	108	4.5
**Partner’s education**		
No education	530	22.4
Primary	578	24.4
Secondary and above	1262	53.3
**Mother’s age**		
15-24 years	997	41.9
25-34 years	1103	46.3
35-49years	281	11.8
**Mother’s age at birth**		
< 20 years	492	20.7
20-29 years	1458	61.3
30-39 years	367	15.4
≥40 years	62	2.6
**Marital status**		
Currently married	2361	99.2
Formerly married^	19	0.8
**Birth order**		
First-born	835	35.1
2nd -4th	1235	51.9
5 or more	310	13.0
**Preceding birth interval**		
No previous birth	835	35.1
< 24 months	324	13.6
> 24 months	1219	51.3
**Place of delivery**		
Home	1560	65.6
Health facility	820	34.4
**Mode of delivery**		
Non-caesarean	2273	95.5
Caesarean	107	4.5
**Combined Place and mode of delivery**		
Home delivery	1560	65.5
Health facility with non-caesarean	713	30.0
Health facility with caesarean	107	4.5
**Type of delivery assistance**		
Health professional	772	32.4
Traditional birth attendant	44	1.9
Relatives and other untrained personnel	1490	62.6
No one	74	3.1
**Antenatal clinic visits**		
None	317	16.8
1-3.	647	34.3
4+	921	48.9
**Timing of postnatal check-up**		
No postnatal check-up	1756	73.8
0-2 days	429	18.0
Delayed	196	8.2
**Currently breastfeeding**		
Yes	1830	76.9
No	550	23.1
**Duration of breastfeeding**		
Up to 12 months	498	20.9
> 12 months	1882	79.1
**Mother’s literacy**		
Can’t read at all	1016	42.7
Can read	1364	57.3
**Child level factors**		
**Sex of baby**		
Male	1208	50.7
Female	1172	49.3
**Perceived size of baby at birth**		
Small	421	17.7
Average	1531	64.5
Large	424	17.8
**Child’s age in months**		
0-5	206	8.8
6-11	240	10.3
12-17	269	11.6
18-23	215	9.2
24-29	227	9.8
30-35	252	10.8
36-41	273	11.7
42-47	230	9.9
48-53	220	9.5
54-59	195	8.4
**Child had diarrhoea recently**		
No	2028	85.2
Yes	352	14.8
**Child had fever in last two weeks**		
No	1883	79.1
Yes	497	20.9
**Household level factors**		
**Household food insecurity (Access)**		
Food secure	1004	43.6
Mildly insecure	129	5.6
Moderately	803	34.9
Severely	365	15.9
**Wealth index**		
Poorest	608	25.6
Poorer	483	20.3
Middle	555	23.3
Rich	406	17.1
Richest	328	13.8
**Source of drinking water**		
Unprotected	409	17.2
Protected	1972	82.8
**Community level factors**		
**Type of residence**		
Urban	211	8.9
Rural	2169	91.1
**Caste group**		
B/C (Hill and Terai)	724	30.4
Newar	63	2.7
Hill Janajati	541	22.8
TeraiJanajati and other Terai castes	469	19.7
Dalit	434	18.2
Muslim and others	149	6.3
**Ecological Zone**		
Mountain	189	7.9
Hill	940	39.5
Terai	1252	52.6
**Geographic Zones**		
Eastern	567	23.8
Central	751	31.6
Western	434	18.2
Mid-western	359	15.1
Far-western	269	11.3
**Sub-Region**		
Eastern Mountain	46	1.9
Central Mountain	42	1.7
Western Mountain	101	4.3
Eastern Hill	174	7.3
Central Hill	215	9.0
Western Hill	274	11.5
Mid-Western Hill	165	6.9
Far-Western Hill	112	4.7
Eastern Terai	348	14.6
Central Terai	495	20.8
Western Terai	160	6.7
Mid-Western Terai	136	5.7
Far-Western Terai	113	4.7

^^^divorced/separated/widowed.

Household food insecurity scores: Food secure (score, 0); mildly insecurity (score, 1–2), moderately (score, 3–10) and severely (score, 10–27).

### Multivariate analyses

Tables 2 and 3 show the unadjusted and adjusted ORs for the association between stunted and severely stunted children by parental-, child-, household- and community-level characteristics of children aged 0–23 and aged 0–59 months.

**Table 2:** **Unadjusted and adjusted odds ratios (OR) (95% CI) for stunted children aged 0–23 and 0–59 months**

Characteristic	Stunted children 0–23 Months				Stunted children 0–59 Months			
	Unadjusted Odd Ratio (OR) [95%CI]	***P***	Adjusted Odd Ratio (AOR) [95%CI]	***P***	Unadjusted Odd Ratio (OR) [95%CI]	***P***	Adjusted Odd Ratio (AOR) [95%CI]	***P***
**Parental factors**								
**Maternal working status**								
Non-working	1.00				1.00			
Working (past 12 months)	1.60 (1.09, 2.34)	0.015			1.30 (0.99, 1.70)	0.057		
**Maternal education**								
No education	1.00				1.00			
Primary	0.75 (0.47,1.18)	0.205			0.77 (0.58, 1.03)	0.079		
Secondary and above	0.39 (0.24, 0.62)	<0.001			0.46 (0.35, 0.60)	<0.001		
**Partner’s occupation**								
Non agriculture	1.00				1.00			
Agriculture	1.80 (1.24,2.63)	0.002			1.68 (1.30, 2.18)	<0.001		
Not working	0.28 (0.07,0.62)	0.071			0.57 (0.29, 1.13)	0.109		
**Partner’s education**								
No education	1.00				1.00			
Primary	0.67 (0.40, 1.11)	0.121			0.91 (0.62, 1.33)	0.620		
Secondary and above	0.40 (0.26, 0.60)	<0.001			0.58 (0.42, 0.79)	0.001		
**Mother’s age**								
15-24 years	1.00				1.00			
25-34 years	1.16 (0.81,1.67)	0.420			1.16 (0.94,1.43)	0.159		
35-49years	3.14 (1.54, 6.01)	0.001			1.91 (1.39, 2.63)	<0.001		
**Mother’s age at birth**								
< 20 years	1.00		1.00		1.00			
20-29 years	0.98 (0.60,1.62)	0.944	1.02 (0.58, 1.80)	0.955	0.91 (0.68, 1.21)	0.508		
30-39 years	1.17 (0.65,2.11)	0.595	0.95 (0.48, 1.87)	0.877	1.06 (0.75, 1.52)	0.728		
40 and above	11.06 (4.26, 28.72)	<0.001	7.31 (2.12, 25.22)	0.002	2.28 (1.27, 4.11)	0.006		
**Marital status**								
Currently married	1.00				1.00			
Formerly married^^^	3.10 (0.26, 36.98)	0.370			1.52 (0.51, 4.52)	0.450		
**Birth order**								
First-born	1.00				1.00			
2nd -4th	1.60 (1.13, 2.25)	0.008			1.25 (1.00, 1.56)	0.052		
5 or more	3.45 (1.91, 6.23)	<0.001			2.15 (1.57, 2.94)	<0.001		
**Preceding birth interval**								
No previous birth	1.00				1.00			
< 24 months	2.20 (1.35, 3.58)	0.002			1.64 (1.22, 2.20)	0.001		
> 24 months	1.77 (1.22, 2.57)	0.003			1.34 (1.08, 1.66)	0.009		
**Type of delivery assistance**								
Health professional	1.00				1.00			
Traditional birth attendant	0.88 (0.19, 4.04)	0.873			1.24 (0.58, 2.64)	0.578		
Relatives or other	3.14 (2.00, 4.94)	<0.001			2.39 (1.87, 3.06)	<0.001		
No one	5.10 (2.16, 12.04)	<0.001			5.63 (3.39, 9.32)	<0.001		
**Combined Place and mode of delivery**								
Home delivery	1.00		1.00		1.00		1.00	
Health facility with non-caesarean	0.33 (0.21,0.51)	<0.001	0.54 (0.33, 0.90)	0.018	0.42 (0.33, 0.52)	<0.001	0.65 (0.51, 0.84)	0.001
Health facility with caesarean	0.22 (0.07, 0.66)	0.007	0.53 (0.16, 1.75)	0.298	0.28 (0.16, 0.50)	<0.001	0.53 (0.29, 0.95)	0.033
**Timing of postnatal check-up**								
No postnatal check-up	1.00				1.00			
0-2 days	0.39 (0.24, 0.63)	<0.001			0.38 (0.30, 0.49)	<0.001		
Delayed	0.45 (0.23, 0.87)	0.019			0.40 (0.26, 0.60)	<0.001		
**Antenatal clinic visits**								
None	1.00				1.00			
1-3.	0.71 (0.36, 1.39)	0.312			0.69 (0.47, 1.01)	0.056		
4+	0.41 (0.23, 0.73)	0.003			0.46 (0.32,0.65)	<0.001		
**Currently breastfeeding**								
Yes	1.00				1.00		1.00	
No	1.02 (0.33, 3.18)	0.971			0.97 (0.75, 1.26)	0.841	0.70 (0.53, 0.94)	0.017
**Duration of breastfeeding**								
Upto 12 months	1.00		1.00		1.00		1.00	
> 12 months	2.80 (1.91, 4.09)	<0.001	2.60 (1.87, 4.02)	<0.001	4.22 (3.12, 5.69)	<0.001	3.54 (2.41, 5.19)	<0.001
**Mother’s literacy**								
Can’t read at all	1.00				1.00			
Can read	0.52 (0.35, 0.76)	0.001			0.58 (0.45, 0.73)	<0.001		
**Child level factors**								
**Child age**								
**Sex of baby**								
Male	1.00				1.00			
Female	0.75 (0.53, 1.06)	0.104			0.93 (0.78, 1.10)	0.392		
**Perceived size of baby at birth**								
Small	1.00		1.00		1.00		1.00	
Average	0.51 (0.32, 0.84)	0.008	0.61 (0.36, 1.04)	0.070	0.64 (0.49, 0.83)	0.001	0.68 (0.51, 0.90)	0.008
Large	0.40 (0.22, 0.75)	0.004	0.42 (0.36, 0.83)	0.013	0.46 (0.33, 0.65)	<0.001	0.47 (0.33, 0.67)	<0.001
**Child’s age in months**	2.39 (1.59, 3.59)	<0.001			1.34 (1.25, 1.44)	<0.001	1.11 (1.01, 1.23)	0.031
**Child had diarrhoea recently**								
No	1.00				1.00			
Yes	1.29 (0.83, 2.02)	0.254			0.91 (0.66, 1.27)	0.589		
**Child had fever in last two weeks**								
No	1.00				1.00			
Yes	1.20 (0.79,1.84)	0.389			0.79 (0.63, 0.98)	0.035		
**Household level factors**								
**Food insecurity (Access)**								
Food secure	1.00				1.00			
Mildly insecurity	0.79 (0.34, 1.82)	0.571			1.11 (0.71, 1.76)	0.638		
Moderately	2.34 (1.48, 3.70)	<0.001			1.66 (1.28, 2.16)	<0.001		
Severely	2.94 (1.54, 5.63)	0.001			2.22 (1.54, 3.20)	<0.001		
**Wealth index**								
poorest	1.00		1.00		1.00		1.00	
poorer	0.79 (0.50, 1.25)	0.314	0.93 (0.58, 1.50)	0.711	0.65 (0.47, 0.88)	0.005	0.67 (0.48, 0.94)	0.020
middle	0.45 (0.28, 0.72)	0.001	0.59 (0.36, 0.97)	0.039	0.42 (0.30, 0.59)	<0.001	0.47 (0.33, 0.68)	<0.001
richer	0.25 (0.14, 0.45)	<0.001	0.31 (0.16, 0.59)	<0.001	0.33 (0.24, 0.46)	<0.001	0.38 (0.25, 0.56)	<0.001
richest	0.16 (0.07, 0.34)	<0.001	0.29 (0.12, 0.67)	0.004	0.26 (0.18, 0.37)	<0.001	0.37 (0.25, 0.55)	<0.001
**Source of drinking water**								
Unprotected	1.00		1.00		1.00			
Protected	1.50 (0.95, 2.36)	0.084			1.08 (0.81, 1.43)	0.609		
**Community level factors**								
**Type of residence**								
Urban	1.00				1.00			
Rural	2.14 (1.26, 3.64)	0.005			1.95 (1.47, 2.58)	<0.001		
**Caste group**								
B/C (Hill and Terai)	1.00				1.00			
Newar	0.72 (0.26, 2.05)	0.542			0.77 (0.40, 1.48)	0.423		
Hill Janajati	1.14 (0.71, 1.85)	0.577			1.35 (0.98, 1.84)	0.063		
TeraiJanajati and other terai castes	1.52 (0.94, 2.43)	0.086			1.11 (0.77, 1.62)	0.566		
Dalit	1.46 (0.92, 2.33)	0.110			1.53 (1.18, 1.99)	0.002		
Muslim and others	0.91 (0.36, 2.26)	0.832			0.80 (0.48, 1.32)	0.382		
**Ecological Zone**								
Mountain	1.00				1.00		1.00	
Hill	0.55 (0.35, 0.87)	0.010			0.63 (0.47, 0.84)	0.002	0.68 (0.50, 0.93)	0.015
Terai	0.48 (0.30, 0.77)	0.002			0.52 (0.39, 0.70)	<0.001	0.80 (0.57, 1.12)	0.193
**Geographic Zones**								
Eastern	1.00				1.00			
Central	1.28 (0.75, 2.17)	0.366			1.04 (0.72, 1.49)	0.832		
Western	1.13 (0.61, 2.10)	0.692			1.07 (0.72, 1.60)	0.738		
Mid-western	1.64 (0.96, 2.77)	0.068			1.71 (1.15, 2.55)	0.008		
Far-western	1.28 (0.73, 2.25)	0.387			1.47 (0.96, 2.24)	0.075		

^^^divorced/separated/widowed.

Household food insecurity scores: Food secure (score, 0); mildly insecurity (score, 1–2), moderately (score, 3–10) and severely (score, 10–27).

**Table 3:** **Unadjusted and adjusted odds ratios (OR) (95% CI) for severely stunted children aged 0–23 and 0–59 months**

Characteristic	Severely stunted children 0–23 Months				Severely stunted children 0–59 Months			
	Unadjusted OR [95%CI]	***p***	Adjusted Odd Ratio (AOR) [95%CI]	***p***	Unadjusted OR [95%CI]	***p***	Adjusted Odd Ratio (AOR) [95%CI]	***p***
**Parental factor**								
**Maternal working status**								
Non-working	1.00				1.00			
Working (past 12 months)	2.10 (1.16, 3.80)	0.015			1.10 (0.78, 1.55)	0.603		
**Maternal education**								
No education	1.00				1.00			
Primary	0.69 (0.34, 1.42)	0.313			0.56 (0.37, 0.84)	0.005		
Secondary and above	0.44 (0.21, 0.91)	0.027			0.35 (0.24, 0.52)	<0.001		
**Partner’s occupation**								
Non agriculture	1.00				1.00			
Agriculture	1.39 (0.77, 2.52)	0.270			1.75 (1.20, 2.54)	0.004		
Not working	0.59 (0.10, 3.54)	0.558			0.40 (0.14, 1.15)	0.088		
**Partner’s education**					1.00			
No education	1.00				0.73 (0.46, 1.15)	0.172		
Primary	0.62 (0.26, 1.47)	0.277			0.43 (0.28, 0.65)	<0.001		
Secondary and above	0.39 (0.19, 0.78)	0.008						
**Mother’s age**								
15-24 years	1.00				1.00			
25-34 years	1.29 (0.71,2.33)	0.406			1.07 (0.77, 1.49)	0.684		
35-49years	4.50 (2.04, 9.94)	<0.001			1.84 (1.28, 2.65)	0.001		
**Mother’s age at birth**								
< 20 years	1.00				1.00			
20-29 years	0.72 (0.36, 1.44)	0.351			0.79 (0.55, 1.14)	0.213		
30-39 years	1.36 (0.60, 3.10)	0.457			1.17 (0.76, 1.79)	0.481		
40 and above	5.85 (1.81, 18.96)	0.003			1.53 (0.76, 3.08)	0.232		
**Marital status**								
Currently married	-'	-'			1.00			
Formerly married^^^	-'	-'			0.76 (0.23, 2.54)	0.653		
**Birth order**								
First-born	1.00				1.00			
2nd -4th	2.08 (1.14, 3.79)	0.017			1.39 (1.01, 1.90)	0.042		
5 or more	4.68 (2.19, 10.00)	<0.001			2.14 (1.39, 3.30)	0.001		
**Preceding birth interval**								
No previous birth	1.00		1.00		1.00			
< 24 months	3.59 (1.75, 7.38)	0.001	2.38 (1.12, 5.03)	0.024	2.25 (1.51, 3.34)	<0.001		
> 24 months	2.21 (1.18, 4.14)	0.014	1.54 (0.80, 2.99)	0.195	1.36 ( 0.97, 1.89)	0.073		
**Type of delivery assistance**								
Health professional	1.00		1.00		1.00		1.00	
Traditional birth attendant	1.56 (0.20, 12.33)	0.674	1.58 (0.19, 13.14)	0.670	1.08 (0.24, 4.82)	0.922	0.65 (0.14, 3.11)	0.589
Relatives or other	3.67 (1.91, 7.03)	<0.001	2.15 (0.98, 4.72)	0.056	2.65 (1.83, 3.83)	<0.001	1.55 (1.05, 2.31)	0.029
No one	7.32 (2.53, 21.22)	<0.001	3.69 (1.14, 11.93)	0.029	7.07 (3.84, 13.01)	<0.001	2.88 (1.47, 5.67)	0.002
**Combined Place and mode of delivery**								
Home delivery	1.00				1.00			
Health facility with non-caesarean	0.33 (0.18,0.60)	<0.001			0.41 (0.29, 0.58)	<0.001		
Health facility with caesarean	0.37 (0.07,1.90)	0.231			0.16 (0.04, 0.59)	0.006		
**Timing of postnatal check-up**								
No postnatal check-up	1.00				1.00			
0-2 days	0.44 (0.23,0.85)	0.014			0.34 (0.23, 0.51)	<0.001		
Delayed	0.33 (0.11, 0.97)	0.045			0.29 (0.14, 0.62)	0.001		
**Antenatal clinic visits**								
None	1.00				1.00			
1-3.	0.72 (0.34,1.49)	0.372			0.77 (0.52, 1.15)	0.201		
4+	0.50 (0.25,1.01)	0.053			0.46 (0.30, 0.71)	0.001		
**Currently breastfeeding**								
Yes	1.00				1.00		1.00	
No	0.86 (0.27, 2.70)	0.789			0.58 (0.41, 0.82)	0.002	0.49 (0.34, 0.69)	<0.001
**Duration of breastfeeding**								
Up to 12 months	1.00		1.00		1.00		1.00	
> 12 months	2.90 (1.81, 5.22)	<0.001	2.87 (1.54, 5.34)	0.001	3.75 (2.30, 6.11)	<0.001	4.15 (2.49, 6.93)	<0.001
**Mother’s literacy**								
Can’t read at all	1.00				1.00		1.00	
Can read	0.55 (0.31, 0.97)	0.039			0.42 (0.31, 0.59)	<0.001	0.61 (0.43, 0.86)	0.005
**Child level factors**								
**Sex of baby**								
Male	1.00		1.00		1.00			
Female	0.46 (0.29, 0.72)	0.001	0.44 (0.28, 0.71)	0.001	0.95 (0.72, 1.26)	0.710		
**Perceived size of baby at birth**								
Small	1.00				1.00		1.00	
Average	0.65 (0.37, 1.14)	0.133			0.70 (0.50, 0.98)	0.038	0.81 (0.57, 1.16)	0.243
Large	0.46 (0.20, 1.07)	0.070			0.43 (0.27, 0.68)	<0.001	0.47 (0.29, 0.74)	0.001
**Child’s age in months**	2.62 (1.41, 4.86)	0.002			1.23 (1.14, 1.33)	<0.001		
**Child had diarrhoea recently**								
No	1.00				1.00			
Yes	1.53 (0.80,2.93)	0.201			1.01 (0.69, 1.47)	0.968		
**Child had fever in last two weeks**								
No	1.00				1.00			
Yes	1.50 (0.85, 2.65)	0.165			0.89 (0.64, 1.23)	0.478		
**Household level factors**								
**Household Food Insecurity (Access)**								
Food secure	1.00				1.00			
Mildly insecurity	1.77 (0.50, 6.34)	0.377			0.85 (0.43, 1.68)	0.637		
Moderately	2.88 (1.27,6.55)	0.012			1.61 (1.06, 2.43)	0.024		
Severely	4.80 (2.00, 11.50)	<0.001			2.41 (1.47, 3.96)	0.001		
**Wealth Index**								
poorest	1.00		1.00		1.00		1.00	
poorer	0.82 (0.42, 1.60)	0.558	0.81 (0.40, 1.66)	0.566	0.76 (0.52, 1.11)	0.156	0.91 (0.61, 1.36)	0.638
middle	0.42 (0.20, 0.87)	0.020	0.49 (0.22, 1.05)	0.067	0.45 (0.30, 0.69)	<0.001	0.60 (0.40, 0.91)	0.016
richer	0.28 (0.11, 0.69)	0.006	0.36 (0.14, 0.93)	0.034	0.33 (0.20, 0.53)	<0.001	0.49 (0.30, 0.82)	0.007
richest	0.19 (0.06, 0.60)	0.005	0.33 (0.10, 1.15)	0.081	0.19 (0.11, 0.33)	<0.001	0.40 (0.20, 0.80)	0.009
**Source of drinking water**								
Unprotected	1.00				1.00			
Protected	0.91 (0.48,1.74)	0.779			0.87 (0.52, 1.24)	0.455		
**Community level factors**								
**Type of residence**								
Urban	1.00				1.00			
Rural	1.62 (0.79, 3.30)	0.187			3.00 (1.86, 4.83)	<0.001		
**Caste group**								
B/C (Hill and Terai)	1.00				1.00			
Newar	0.84 90.22,3.25)	0.796			0.82 (0.31, 2.13)	0.682		
Hill Janajati	0.97 (0.45, 2.09)	0.935			1.25 (0.87, 1.80)	0.233		
TeraiJanajati and other terai castes	0.99 (0.38, 2.64)	0.997			1.30 (0.72, 2.38)	0.385		
Dalit	1.35 (0.68, 2.69)	0.389			1.74 (1.24, 2.44)	0.001		
Muslim and others	0.69 (0.26, 1.87)	0.468			0.92 (0.46, 1.85)	0.825		
**Ecological Zone**								
Mountain	1.00				1.00			
Hill	0.81 (0.39, 1.68)	0.565			0.69 (0.47, 1.01)	0.059		
Terai	0.60 (0.28, 1.29)	0.191			0.63 (0.42, 0.94)	0.025		
**Geographic Zones**								
Eastern	1.00				1.00			
Central	1.46 (0.64, 3.30)	0.363			1.37 (0.86, 2.20)	0.184		
Western	2.13 (0.91, 5.03)	0.083			1.23 (0.72, 2.09)	0.449		
Mid-western	2.41 (1.16, 5.00)	0.018			1.77 (1.12, 2.81)	0.015		
Far-western	1.70 (0.68, 4.25)	0.254			1.46 (0.85, 2.52)	0.168		

^^^divorced/separated/widowed.

Household food insecurity scores: Food secure (score, 0); mildly insecurity (score, 1–2), moderately (score, 3–10) and severely (score, 10–27).

#### Risk factors for stunting

As shown in Table 2, children aged 0–23 months delivered by older mothers (adjusted OR = 7.36, 95%CI: 2.11, 25.75; p = 0.002 for mothers aged 40 and above) were significantly more likely to be stunted than those delivered by younger mothers (mothers less than 20 years old).

For children aged 0–23 months, those who were delivered at the health facility by non-caesarean section (adjusted OR = 0.55, 95%CI: 0.33, 0.92; p = 0.022) were significantly less likely to be stunted compared with children delivered at home. Babies who were perceived to be large by their mothers were 58% less likely to be stunted than those who were perceived to be small (adjusted OR = 0.42, 95%CI: 0.22, 0.81; p = 0.010 for large babies). Also, children aged 0–23 months who were breastfed for up to 12 months were significantly less likely to be stunted than those breastfed for more than 12 months.

Children aged 0–23 months from middle-income households (adjusted OR = 0.53, 95%CI: 0.33, 0.85; p = 0.009), those from richer households (adjusted OR = 0.28, 95%CI: 0.15, 0.53; p < 0.001) and those from richest households (adjusted OR = 0.26, 95%CI: 0.11, 0.60; p = 0.002) were significantly less likely to be stunted compared to those from poorest households. Children aged 0–23 months who had no access to protected drinking water were 1.74 times more likely to be stunted than those who had access to protected drinking water. In the final model, we removed household wealth index and replaced with father’s education. The result indicated that children aged 0–23 months whose fathers attained secondary education or higher were 44% less likely to be stunted compared with children whose fathers had no formal education (adjusted OR = 0.56; CI: 0.37, 0.86; p = 0.007 for fathers with secondary education or higher).

Children aged 0–59 months who were currently being breastfed were significantly less likely to be severely stunted compared with children of the same age group who were not currently being breastfed (adjusted OR = 0.70; CI: 0.54, 0.94; p = 0.017); and children aged 0–59 months who breastfed for more than 12 months were more likely to be stunted than those breastfed for up to 12 months. Children aged 0–59 months who were perceived by their mothers to be average size (adjusted OR = 0.68, 95%CI: 0.51, 0.90; p = 0.008) and those perceived to be large (adjusted OR = 0.47, 95%CI: 0.33, 0.67; p < 0.001) at the time of delivery were significantly less likely to be stunted than children of the same age perceived to be small at the time of delivery.

Children aged 0–59 months from poorer households (adjusted OR = 0.67, 95%CI: 0.48, 0.94; p = 0.020), middle-income households (adjusted OR = 0.47, 95%CI: 0.33, 0.68; p < 0.001), and richer households (adjusted OR = 0.38, 95%CI: 0.25, 0.56; p < 0.001) and those from richest households (adjusted OR = 0.37, 95%CI: 0.25, 0.55; p < 0.001) were significantly less likely to be stunted compared with those from poorest households. Increasing age of the child was significantly associated with stunting (adjusted OR = 1.11, 95%CI: 1.01, 1.23; p = 0.031) andchildren aged 0–59 months from the Hill zone (adjusted OR = 0.68, 95%CI: 0.50, 0.93; p = 0.015) were significantly less likely to be stunted compared with those who lived in the Mountains. In the final model for stunted children aged 0–59 months, we removed household wealth index and replaced it with household food security and our result revealed that households who reported moderate and severe food insecurity were 1.37 times and 1.67 times more likely to be stunted than those who reported food security (adjusted OR = 1.37, 95%CI: 1.02, 1.85; p = 0.039 for moderately food insecure households and adjusted OR = 1.67, 95%CI: 1.17, 2.38; p = 0.005) for severely food insecure households).

#### Risk factors for severe stunting

Table 3 illustrates the unadjusted and adjusted odds ratios for the association between severely stunted children and parental-, child-, household- and community-level factors of children aged 0–23 months and 0–59 months. Girls aged 0–23 months had statistically significantly reduced odds of being severely stunted compared to boys aged 0–23 months (AOR = 0.44, 95% CI: 0.28, 0.71; p = 0.001). Children aged 0–23 months from rich household had reduced odds of being severely stunted (AOR = 0.36, 95%CI: 0.14, 0.93; p = 0.034) compared with those from poorest household. Children aged 0–23 months who were delivered without assistance to their mothers and mothers of children with preceding birth interval less than 24 months were significantly more likely to be severely stunted than those children delivered by health professional and those with mothers with no previous birth. In the final model for severely stunted children aged 0–23 months, when household wealth index was removed and replaced with household food security, the result indicated that household who reported severe food insecurity were 3.27 times more likely to be severely stunted than those who reported food security (adjusted OR = 3.27, 95%CI: 1.30, 8.20; p = 0.016).

Children aged 0–59 months who were delivered with assistance by relatives or others and those delivered with no assistance were significantly more likely to be stunted compared with children who were delivered with assistance from a health professional. The odds for severely stunted children aged 0–59 months for babies not currently being breastfed and children whose mothers could not read decreased significantly by 41% (adjusted OR = 0.49; CI: 0.34, 0.69; p < 0.001 for currently being breastfed children aged 0–59 months) and 51% (adjusted OR = 0.49; CI: 0.34, 0.69; p < 0.001 for children whose mothers could read). Children aged 0–59 months who were breastfed for more than 12 months (adjusted OR = 4.15, 95%CI: 2.49, 6.93; p < 0.001) were significantly more likely to be severely stunted than those children aged 0–59 months who were breastfed for up to 12 months.

Children aged 0–59 months perceived by their mothers to be large (adjusted OR = 0.47, 95%CI: 0.33, 0.67; p = 0.001) were significantly less likely to be stunted than children of the same group perceived to be small by their mothers at the time of delivery. Children aged 0–59 months from middle-income households (adjusted OR = 0.60, 95%CI: 0.40, 0.91; p = 0.016), richer households (adjusted OR = 0.49, 95%CI: 0.30, 0.82; p = 0.007) and those from richest households (adjusted OR = 0.40, 95%CI: 0.20, 0.80; p = 0.009) were significantly less likely to be severely stunted than those children aged 0–59 months from poorest households. In the final model for severely stunted children aged 0–59 months, when household wealth index was removed and replaced with type of residence, we observed that children aged 0–59 months who lived in rural areas were more likely to be severely stunted than their urban counterparts (adjusted OR = 1.99, 95%CI: 1.23, 3.25; p = 0.006 for rural residence).

## Discussion

This paper presents the risk factors for stunting and severe stunting among children aged 0–23 and 0–59 months using the 2011 NDHS data. The findings from this study would enable public health researchers to reshape and redesign new educational interventions to reduce the prevalence of stunting in Nepal. The prevalences of stunting and severe stunting in children less than 23 months of age were as high as the global estimate of 27% [18] while the prevalences of stunting and severe stunting in children aged 0–59 months were also high (NDHS, 2011) but slightly lower than those of Bangladesh and India [19, 20]. Despite many interventions to reduce the level of stunting among under-five children in Nepal, over the past 10 years, the prevalence remains consistently high [8]. This explains the fact that there are other underlying factors contributing to the high rate of stunting among children aged less than five years. However, the reported prevalence of stunting and severe stunting among children aged under five years in Nepal was within the highest range (40-58%) reported among other 20 developing countries [21].

This study indicated that increasing age of the child was significantly associated with stunting and severe stunting and children aged 0–23 months significantly reported a lower risk of stunting and severe stunting than those in the older age group of 0–59 months. Similar results were found by other researchers [22, 23]. The finding could be explained by the protective effect of breastfeeding as most children in Nepal are breastfed even into the second year of life [24]. The high rate of stunting and severe stunting observed among children 0–59 months may be associated with inappropriate food supplementation during the weaning period [24, 25].

This study revealed that breastfed children for more than 12 months were significantly more likely to be stunted and severely stunted than those breastfed for up to 12 months, which indicated that stunting and severe stunting correlated with prolonged duration of breastfeeding. These findings support the study that stunting occurs most readily in the first 6–18 months [26]. Another study [27] found that stunting was most common among children aged 36–47 months (51.89%) followed by 12–23 age groups (50.64%) and it was lowest in the older age group of 48–59 months (39.13%). These variations could be linked to other contributing factors such as culture, exclusive breastfeeding status, time of initiation of complementary feeding, socioeconomic dynamics and parents’ educational status in that community [24, 25].

Another risk factor for stunting and severe stunting in this age group was household wealth index. Our study revealed that children from poorest households were more likely to become stunted or severely stunted compared to those from middle-income, richer and richest households. This finding suggests that a child’s health status depends upon the socio-economic standing of their household. Also, educated mothers who are more conscious about their children’s health and nutritional needs are most likely to come from richer households. Previous studies among Peruvian, Cambodian and Bangladeshi children found household wealth index to be a key predictor for stunting and severe stunting among children under five years of age [25, 28–30]. The association between low income and stunting has been observed in several other studies [25, 29, 31–33]. Rich households have greater purchasing power for food and other consumer goods needed to ensure the health of children. Such children are therefore not likely to be exposed to conditions that would lead to stunting or severe stunting.

Our study also revealed that children perceived by their mothers to be small had a higher risk of being stunted compared to those perceived to be average or large. These findings were supported by studies previously conducted in Pakistan and Mexico [34, 35] which indicated that children less than 24 months of age with lower birth weight were 3 times more likely to be stunted than children of the same age group with normal or higher birth weight. As the incidence of low birth weight (<2.5 kg) is high (21%) in Nepal [21], prevention of intrauterine growth retardation, premature delivery and maternal malnutrition should be one of the basis in public health level intervention strategy for infant stunting. The assessment of the baby’s size at birth by health-care providers could be significant in identifying children at risk of stunting. In our analysis, maternal age at child’s birth was found to be an important risk factor for childhood stunting and severe stunting. Children in the age group 0–23 months born to younger mothers (aged <20 years) were less likely to be stunted compared with those born to older mothers (aged >20 years). These results were consistent with a study conducted in Iran [36]. However, in the Iran study, it was found that children born to mothers older than 35 years of age were more likely to be stunted and severely stunted. On the contrary, a study in Mexico [35] found that maternal age at child’s birth was not a predictor for stunting. These discrepancies in findings could be attributed to differences in cultures, socioeconomic dynamics and nutritional factors among the various communities, as the studies were conducted in different continents of the world.

Among children aged 0–59 months, the type of delivery assistance received was found to be a significant risk factor for stunting. Children who were delivered with assistance from traditional birth attendants or relatives and those who were delivered without any assistance were significantly more likely to be stunted compared to those who were delivered with assistance from health professionals. This finding is supported by a study conducted in India [37] in which children delivered at home were more likely to be stunted compared to those delivered at a health facility. In another study conducted in Bangladesh [33], it was reported that the place of delivery and the assistance received were significantly associated with stunting and severe stunting among pre-school children. These findings could be explained by the health information given to mothers by health professionals during antenatal and postpartum periods. Possible health information that health professionals could offer to mothers may include information on exclusive breastfeeding, initiation of complementary feeding as well as comprehensive care for the new-borns. Further analysis revealed that 78% of mothers who go for institutional delivery are those from richest wealth quintiles compared with 22% of mothers from richest wealth quintiles who delivered their babies at home.

Our study also found that breastfed children were significantly less likely to be stunted compared to non-breastfed children. Also, children born to mothers who could read were significantly less likely to be stunted compared to those born to mothers who could not read at all. This reflects the importance of mother education and breastfeeding in the development of healthy children and has been reported in previous studies [30, 31, 33]. We also found that children who were breastfed and born to educated mothers were less likely to be stunted compared to those who were not breastfed and born to uneducated mothers.

In addition, our study showed that children resident in the Hill zone were significantly less likely to be severely stunted compared to those from the Mountains. This finding is consistent with studies conducted in Bangladesh [32, 33] in which the region where a child was born played a significant role in predicting stunting. This association could be due to the nature of dietary intake, access to food and cultural diversity in that environment.

Replacing household wealth with household food insecurity and type of residence in the final model, this study found a strong association between household food insecurity and stunting and severe stunting among children aged 0–59 months and 0–23 months, respectively. These findings were consistent with a study carried out in Colombia [38] which indicated that household food insecurity was significantly associated with stunting among pre-school children. Our study also found that children aged 0–59 months who lived in the rural areas were significantly more likely to be stunted compared to their urban counterparts. A two-stage cluster study carried out in Vietnam [23] found that living in rural areas was a risk factor for malnutrition including stunting. This study also revealed that rich families were more likely to report food security and also more likely to reside in urban areas.

As the rate of stunting and severe stunting are still high in Nepal, program intervention strategies targeting long-term prevention of stunting in this country are needed to effectively and sustainably improve their prevalence. Education of mothers and improvement of household incomes should be given special attention. This is because children born to uneducated mothers and from poor households have been found to have increased risk of stunting. In general, our findings are of major significance because they identify potential areas for action plans that could improve and sustain the nutritional status of children under-five years of age.

One potential limitation of the study as a secondary data analysis was that, there was no information on dietary habits or insufficient dietary practices to support stunted and severely stunted children. Another limitation was the indirect measure of household wealth in a developing country such as Nepal. It is difficult to obtain consistent income and expenditure data in this country; however, an asset-based index is generally considered a decent proxy for household wealth status.

The sampling method, appropriate adjustment for sampling design, including sampling weight and a high response rate (98%) from the survey are important strengths of this study. The study also contributes to the understanding of the factors associated with stunting and severe stunting among children 0–59 months in Nepal by using the recent 2011 Demographic and Health Survey data. For a developing country like Nepal, this study provides a foundation for planning of intervention strategies to prevent stunting in children less than five years of age. Interventional studies aimed at examining the impact of child and maternal under-nutrition are needed in Nepal and such studies should target mothers from low socioeconomic backgrounds.

## Conclusions

Our analysis of factors associated with stunting and severe stunting among children 0–59 months in Nepal revealed that the common increased risk factors for stunting were combined place and mode of delivery (home delivery), prolonged breastfeeding (more than 12 months), perceived size of baby (small babies), household wealth (poorest households) while types of delivery assistance (mothers delivered by no one), prolonged breastfeeding (more than 12 months), perceived size of baby (small babies), household wealth (poorest households) reported consistenthigh risk factors for severe stunting.Our findings highlight the need for early community-based educational interventions aimed at improving the nutritional status of children underfive years of age in order to achieve optimal brain development and reduce mortality triggered by malnutrition.
